# Incidence and Associated Factors of Anemia in Patients with Acute Moderate and Severe Traumatic Brain Injury

**DOI:** 10.1007/s12028-022-01561-9

**Published:** 2022-08-02

**Authors:** Heidi Vanhala, Eija Junttila, Anneli Kataja, Heini Huhtala, Teemu Luostarinen, Teemu Luoto

**Affiliations:** 1 Department of Anesthesia and Intensive Care, Tampere University Hospital, Tampere, Finland; 2 Medical Imaging Center, Department of Radiology, Tampere University Hospital, Tampere, Finland; 3 Faculty of Social Sciences, Biostatistics Group, Tampere University, Tampere, Finland; 4 Division of Anesthesiology, Department of Anesthesiology, Intensive Care, and Pain Medicine, University of Helsinki and Helsinki University Hospital, Helsinki, Finland; 5 Department of Neurosurgery, Tampere University Hospital and Tampere University, Tampere, Finland

**Keywords:** Traumatic brain injury, Anemia, Critical care

## Abstract

**Background:**

Anemia might contribute to the development of secondary injury in patients with acute traumatic brain injury (TBI). Potential determinants of anemia are still poorly acknowledged, and reported incidence of declined hemoglobin concentration varies widely between different studies. The aim of this study was to investigate the incidence of severe anemia among patients with moderate to severe TBI and to evaluate patient- and trauma-related factors that might be associated with the development of anemia.

**Methods:**

This retrospective cohort study involved all adult patients admitted to Tampere University Hospital’s emergency department for moderate to severe TBI (August 2010 to July 2012). Detailed information on patient demographics and trauma characteristics were obtained, including data on posttraumatic care, data on neurosurgical procedures, and all measured in-hospital hemoglobin values. Severe anemia was defined as a hemoglobin level less than 100 g/L. Both univariate and multivariable analyses were performed, and hemoglobin trajectories were created.

**Results:**

The study included 145 patients with moderate to severe TBI (male 83.4%, mean age 55.0 years). Severe anemia, with a hemoglobin level less than 100 g/L, was detected in 66 patients (45.5%) and developed during the first 48 h after the trauma. In the univariate analysis, anemia was more common among women (odds ratio [OR] 2.84; 95% confidence interval [CI] 1.13–7.15), patients with antithrombotic medication prior to trauma (OR 3.33; 95% CI 1.34–8.27), patients with cardiovascular comorbidities (OR 3.12; 95% CI 1.56–6.25), patients with diabetes (OR 4.56; 95% CI 1.69–12.32), patients with extracranial injuries (OR 3.14; 95% CI 1.69–12.32), and patients with midline shift on primary head computed tomography (OR 2.03; 95% CI 1.03–4.01). In the multivariable analysis, midline shift and extracranial traumas were associated with the development of severe anemia (OR 2.26 [95% CI 1.05–4.48] and OR 4.71 [95% CI 1.74–12.73], respectively).

**Conclusions:**

Severe anemia is common after acute moderate to severe TBI, developing during the first 48 h after the trauma. Possible anemia-associated factors include extracranial traumas and midline shift on initial head computed tomography.

## Introduction

### Background

The intensive care of patients with acute traumatic brain injury (TBI) aims to provide adequate perfusion and oxygen delivery to neuronal tissue, thus avoiding secondary damage to the injured brain. Oxygen supply depends on cerebral blood flow and oxygen-carrying capacity of circulating blood, which in turn derives from the blood hemoglobin (Hb) level and its oxygen saturation. A compromised Hb level activates multiple changes in the circulatory system to compensate for the oxygen deficit [1, 2]. In several animal models, hemodilutional, normovolemic anemia seemed to increase cerebral blood flow [3–5] and intracranial pressure [6], impair cerebrovascular responses to altering CO_2_ levels [7], and lead to cerebral ischemia and worse cerebral injury [8]. In a mathematical model of anemia in focal stroke in rabbits, oxygen uptake by ischemic penumbra decreased progressively when the Hb level decreased to less than 100 g/L [9]. In healthy human volunteers, hemodilutional anemia, with an Hb level less than 70 g/L, resulted in cognitive dysfunction and memory deficits [10]. Because of altered autoregulation [11] and other pathophysiological changes, the injured brain might be more susceptible to anemia and related hypoxemia and might not tolerate the same levels of anemia as noninjured brain tissue [2, 12, 13].

The reported incidence of anemia among patients with TBI varies widely from 37 to 69%, at least partly because of methodological differences between studies [14–17]. Low Hb concentration might be associated with worse outcome [15, 17–19], especially among patients with compromised brain tissue oxygen tension [20]. Data on possible determinants of anemia in patients with TBI are scarce and somewhat contradictory. According to a relatively recent meta-analysis regarding red blood cell transfusion (RBCT) in patients with TBI, age, sex, and injury severity score were not associated with the need for RBCT. In turn, patients receiving RBCT had lower Glasgow Coma Scale scores at admission. In the same meta-analysis, the length of stay (LOS) in the intensive care unit (ICU) was shorter among patients with higher Hb values [21].

### Study Objectives

The primary objective of this study was to evaluate the incidence of anemia and pattern of decline in Hb concentration during hospital stay in patients with acute moderate to severe TBI. The secondary objective was to investigate potential patient- or trauma-related risk factors for post-TBI anemia. Our hypothesis was that the severity of the head trauma, presence of extracranial traumas, and the use of antithrombotic medication, and thus cardiovascular comorbidities, would be associated with the incidence of anemia. In addition, we assumed that regular selective serotonin reuptake inhibitor (SSRI) and serotonin–norepinephrine reuptake inhibitor (SNRI) medications, with their potential adverse effects in primary hemostasis and platelet adhesion, could increase the incidence of anemia.

## Methods

### Study Design and Setting

This study is a retrospective cohort study and a part of the Tampere Traumatic Head and Brain Injury Study. The study was conducted at Tampere University Hospital between August 2010 and July 2012. Tampere University Hospital is a tertiary care hospital and the only neurosurgical referral hospital in the hospital district, providing health services and demanding specialized care for a total of approximately 900,000 residents from 52 municipalities, both urban and rural.

The study population included all consecutive patients assessed at the emergency department of Tampere University Hospital for head trauma and referred to head computed tomography (CT) scanning. Each admission for a new head trauma was recorded as its own case. The minimum criteria for TBI were based on the World Health Organization’s (WHO’s) Collaborating Centre for Neurotrauma Task Force on Mild Traumatic Brain Injury definition presented in 2004 [22]. The Department of Veterans Affair/Department of Defense guideline’s definition of moderate and severe TBI was applied to classify the moderate and severe TBI cases [23]. Additionally, the following exclusion criteria were used: (1) no traumatic intracranial lesions in primary CT, (2) age younger than 18 years at the date of trauma, (3) LOS in the university hospital less than 48 h for any reason, and (4) withdrawal of life-sustaining therapy after the first assessment in the emergency department.

In this study, anemia was defined by the WHO Department of Nutrition for Health and Development recommendations for the diagnosis of anemia [24]. Accordingly, Hb concentration for anemia was set to < 130 g/L for men and < 120 g/L for women. The Hb concentration threshold for RBCTs among patients with acute TBI is traditionally considered to be 90–100 g/L [1, 2]. An Hb concentration threshold of 100 g/L is also the threshold for a liberal transfusion strategy in the HEMOTION (HEMOglobin transfusion threshold in Traumatic brain Injury Optimization) Trial, a randomized prospective clinical trial evaluating the effects of RBCT thresholds on neurological functional outcome [25]. Hence, this Hb concentration threshold was of special interest and was applied to our study population to evaluate the incidence and associated factors of severe anemia.

### Main Variables and Data Sources

Data were obtained retrospectively from medical records and the Tampere Traumatic Head and Brain Injury Study database, with no direct contact to patients or their representatives. The main study variables included patient demographics, injury-related findings, and major comorbidities (e.g., chronic cardiovascular, pulmonary, kidney, or liver diseases; chronic bleeding disorders; and prior TBIs). Also, information on regular medications with effects on blood coagulation and clot formation was obtained, including the use of anticoagulants (warfarin and direct oral anticoagulants), antiplatelet medication (aspirin and adenosine diphosphate receptor inhibitors), SSRIs, SNRIs, and long-term oral corticosteroids. All the head CT scans were interpreted by a neuroradiologist, and the findings were coded in compliance with the Common Data Elements by the National Institute of Neurological Disorders and Stroke [26]. From the in-hospital period, information on major neurosurgical operations (including craniotomy, hemicraniectomy, trepanation, or repairment of skull fractures), duration of ICU and ward admission, and hospital-acquired infections was obtained.

To investigate the incidence of anemia, each measured Hb value, with a corresponding time label, was collected until hospital discharge, interinstitutional transfer, or death or for a maximum of 30 days. Hb values from both blood count samples and arterial blood gas analysis samples were considered.

### Statistical Methods

Variable distributions were defined with the Kolmogorov–Smirnov and Shapiro–Wilk test. For descriptive data, mean with standard deviation (SD) was used with normally distributed variables, and median with interquartile range was used with nonnormally distributed data. The independent-samples *t*-test for normally distributed variables and Mann–Whitney *U*-test for nonnormally distributed variables were used. The *χ*^2^ test was performed for categorial variables. Univariate and multivariable analyses were performed with a binary logistic regression model. Variables for the multivariable analysis were selected on the basis of clinical relevance and in an attempt to avoid overlapping of subgroups, prior to any analysis, and included age, sex, overall comorbidity, the use of any antithrombotic medication, chronic alcohol consumption, presence of extracranial injuries, and midline shift on primary head CT as a surrogate to severe space-occupying brain trauma.

All statistical analyses were performed with IBM SPSS Statistics (Armonk, NY) versions 25 and 27. For all analyses, a *p* value < 0.05 was considered statistically significant.

### Study Ethics

Because this was a retrospective, medical-record-based study, written informed consent from the patients was not required. The primary Tampere Traumatic Head and Brain Injury Study was approved by the Ethical Committee of Pirkanmaa Hospital District, Tampere, Finland (ethics code: R10027). Institutional ethics and research board approval was also obtained.

## Results

### Study Population

**Fig. 1:**
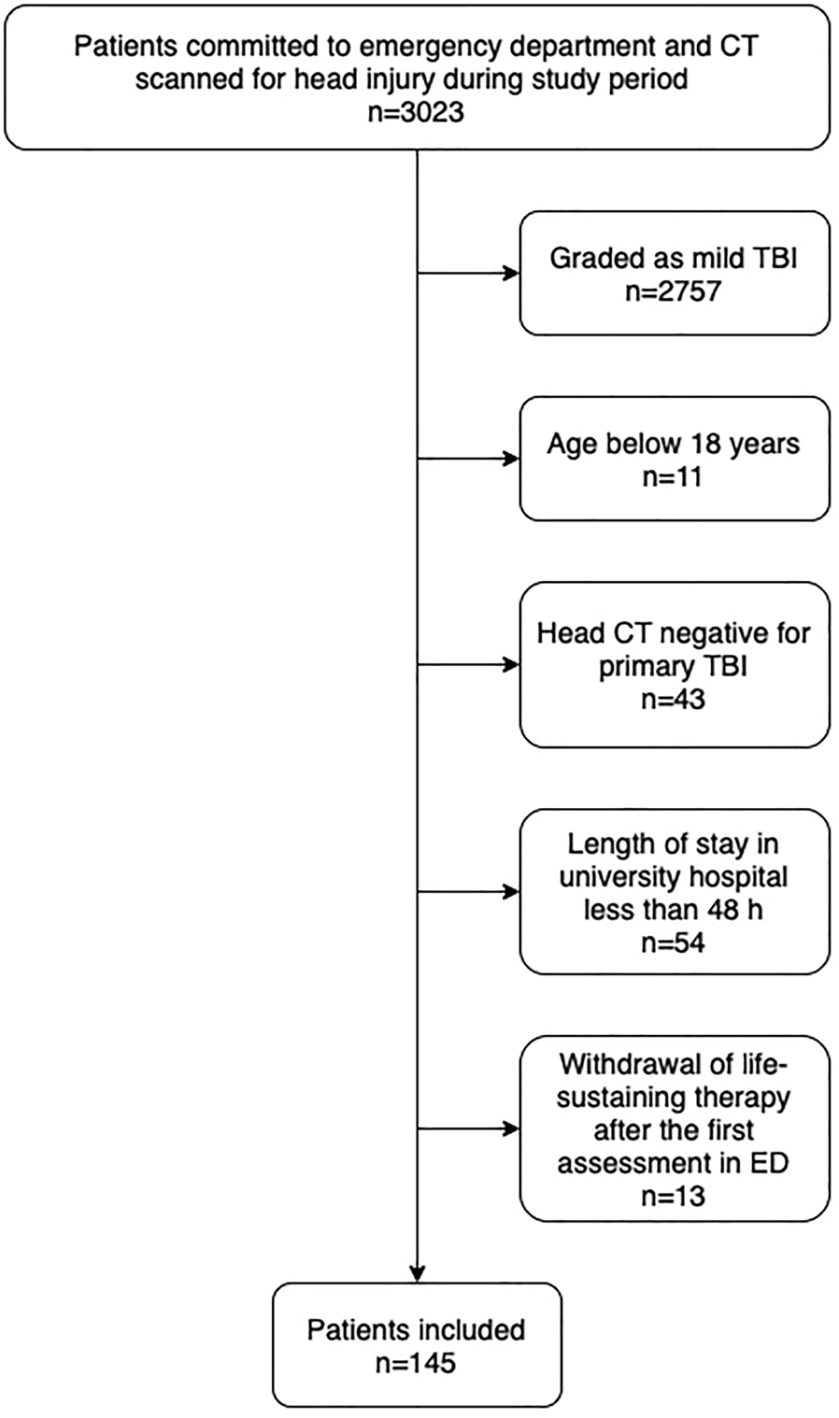
Flowchart of the study. *CT* computed tomography, *ED* emergency department, *TBI* traumatic brain injury

During the study period (August 2010 to July 2012), a total of 3023 patients underwent cranial CT scanning in the emergency department for suspected or confirmed TBI. Of the whole sample, 266 patients had moderate to severe TBI. After the assessment of exclusion criteria, 145 patients were included to this study. The flowchart of the study is presented in Fig. 1.

### Descriptive Data

Of the 145 patients included to the study, 121 (83.4%) were male. The mean age at the point of trauma was 55.0 years (range 18.0–92.4, SD 17.5), with men being significantly younger than women (53.0 vs. 64.7 years, respectively; 95% confidence interval [CI] 4.2–19.2; *p* = 0.003). Major somatic long-term comorbidity (i.e., cardiovascular or pulmonary diseases, diabetes, hepatic cirrhosis, chronic kidney disease, chronic autoimmune disease, or inherited or acquired bleeding disorder) was previously diagnosed in 74 (51.0%) patients, and 50 (34.5%) were chronic alcohol misusers. Prior TBI, either before or during the study period (August 2010 to July 2012), was diagnosed in 22 (15.2%) patients. Of the 145 patients, 117 (80.7%) had an isolated TBI and 131 (90.3%) were admitted to the ICU. The median LOS in the ICU was 2 days (interquartile range 1–5 days), and the median LOS in Tampere University Hospital was 6 days (4–10 days). Patient demographics are presented in Table 1.

**Table 1:** Patient demographics in respect to the development of severe anemia (Hb level less than 100 g/L at any point of hospital admission)

	Hb level ≥ 100 (*n* = 79)	Hb level < 100 (*n* = 66)
**Patient-associated factors**		
Age, years, mean (SD)	52.7 (17.2)	57.7 (17.6)
Male, n (%)	71 (89.9)	50 (75.8)
Major comorbidity, *n* (%)	32 (40.5)	42 (63.6)
Cardiovascular	21 (26.6)	35 (53.0)
Diabetes	6 (7.6)	18 (27.3)
Pulmonary	10 (12.7)	7 (10.6)
Antithrombotic agents, *n* (%)	8 (10.1)	18 (27.3)
Anticoagulant agents	6 (7.6)	10 (15.2)
Antiplatelet agents	2 (2.5)	8 (12.1)
SSRI or SNRI medication, *n* (%)	3 (3.8)	5 (7.6)
Chronic alcohol misuse, *n* (%)	28 (35.4)	22 (33.3)
Previous TBI, *n* (%)	12 (15.2)	10 (15.2)
**Trauma-associated factors**		
Primary GCS score, median (IQR)	11 (6–13)	9 (3–13)
Primary CT findings, n (%)		
Acute subdural hematoma	58 (73.4)	48 (72.7)
Subarachnoid hemorrhage	51 (64.6)	40 (60.6)
Epidural hematoma	8 (10.1)	5 (7.6)
Intra-axial trauma lesions	42 (53.2)	42 (63.6)
Contusions	42 (53.2)	42 (63.6)
DAI	1 (1.3)	3 (4.5)
Midline shift	24 (30.4)	31 (47.0)
Extracranial injuries, *n* (%)	9 (11.4)	19 (28.8)
**Treatment-associated factors**		
Major neurosurgical intervention, n (%)	28 (35.4)	50 (54.8)

*CT* computed tomography, *DAI* diffuse axonal injury, *GCS* Glasgow Coma Scale, *Hb* hemoglobin, *IQR* interquartile range, *SD* standard deviation, *SNRI* serotonin and noradrenalin reuptake inhibitor, *SSRI* selective serotonin reuptake inhibitor, *TBI* traumatic brain injury

### Outcome Data and Main Results

According to the WHO criteria, anemia at any point during hospitalization for acute TBI was detected in 127 (87.5%) patients. Severe anemia (Hb value less than 100 g/L) was diagnosed in 66 patients (45.5%), of whom seven (10.6%) had anemia at admission, and the rest developed Hb concentration decline during the hospital stay. The mean Hb level at admission was 131.5 g/L (SD 18.4), and it was significantly lower in patients who developed severe anemia compared with those who did not (121.3 and 140.1 g/L, respectively; *p* < 0.001). The mean decrease in Hb concentration during the hospital stay was 27.0 g/L (SD 15.3), with a mean proportional decrease of 20.1 percentage points (SD 10.3). The mean decrease in Hb values was significantly greater among anemic patients (absolute decrease 32.4 vs. 22.5 g/L, mean difference − 9.9 [95% CI − 14.7 to − 5.1], *p* < 0.001; proportional decrease 25.5 vs. 15.7 percentage points, mean difference − 9.9 [95% CI − 12.9 to − 6.9], *p* < 0.001). Accordingly, Hb levels less than 90, 80, and 70 g/L were detected in 29 (20.0%), 7 (4.8%), and 3 (2.1%) patients.

The detailed results of univariate and multivariable analyses are presented in Table 2. In the univariate analysis, female sex, cardiovascular comorbidities, diabetes, and the use of antithrombotic agents were associated with the development of severe anemia. Also, the presence of extracranial injuries, midline shift on primary CT, and major neurosurgical procedures were associated with declined Hb level. No association between anemia and SSRI or SNRI medication was found. In the multivariable analysis, only midline shift on primary CT and presence of extracranial injuries were associated with development of severe anemia (odds ratio [OR] 2.26 [95% CI 1.05–4.84] and OR 4.71 [95% CI 1.74–12.73]). Major comorbidity was partly related to the development of severe anemia; however, the result was statistically nonsignificant (OR 2.24, 95% CI 0.93–5.38).

**Table 2:** Results of univariate and multivariable analyses

	Univariate			Multivariable		
	OR	95% CI	*p*	OR	95% CI	*p*
**Patient-associated factors**						
Age	1.02	0.998–1.04	0.085	1.00	0.97–1.02	0.83
Female sex	2.84	1.13–7.15	0.027	2.45	0.86–6.99	0.095
Major comorbidity	2.57	1.31–5.04	0.006	2.24	0.93–5.38	0.072
Cardiovascular	3.12	1.56–6.25	0.001			
Diabetes	4.56	1.69–12.32	0.003			
Pulmonary	0.82	0.29–2.29	0.702			
Antithrombotic agents	3.33	1.34–8.27	0.010	1.94	0.61–6.17	0.264
Anticoagulant agents	2.17	0.75–6.34	0.160			
Antiplatelet agents	5.31	1.09–25.95	0.039			
SSRI or SNRI medication	2.08	0.48–9.04	0.330			
Chronic alcohol misuse	0.91	0.46–1.81	0.790	1.41	0.63–3.15	0.404
Previous TBI	1.00	0.40–2.48	0.995			
**Trauma-associated factors**						
Primary GCS score	0.94	0.85–1.03	0.188			
Primary CT findings						
Intra-axial trauma lesions	1.54	0.79–3.01	0.204			
Midline shift	2.03	1.03–4.01	0.042	2.26	1.05–4.84	0.036
Extracranial injuries	3.14	1.31–7.54	0.010	4.71	1.74–12.73	0.002
**Treatment-associated factors**						
Major neurosurgical intervention	5.69	2.75–11.78	< 0.001			

*CI* confidence interval, *CT* computed tomography, *GCS* Glasgow Coma Scale, *OR* odds ratio, *SNRI* serotonin and noradrenalin reuptake inhibitor, *SSRI* selective serotonin reuptake inhibitor, *TBI* traumatic brain injury

**Fig. 2:**
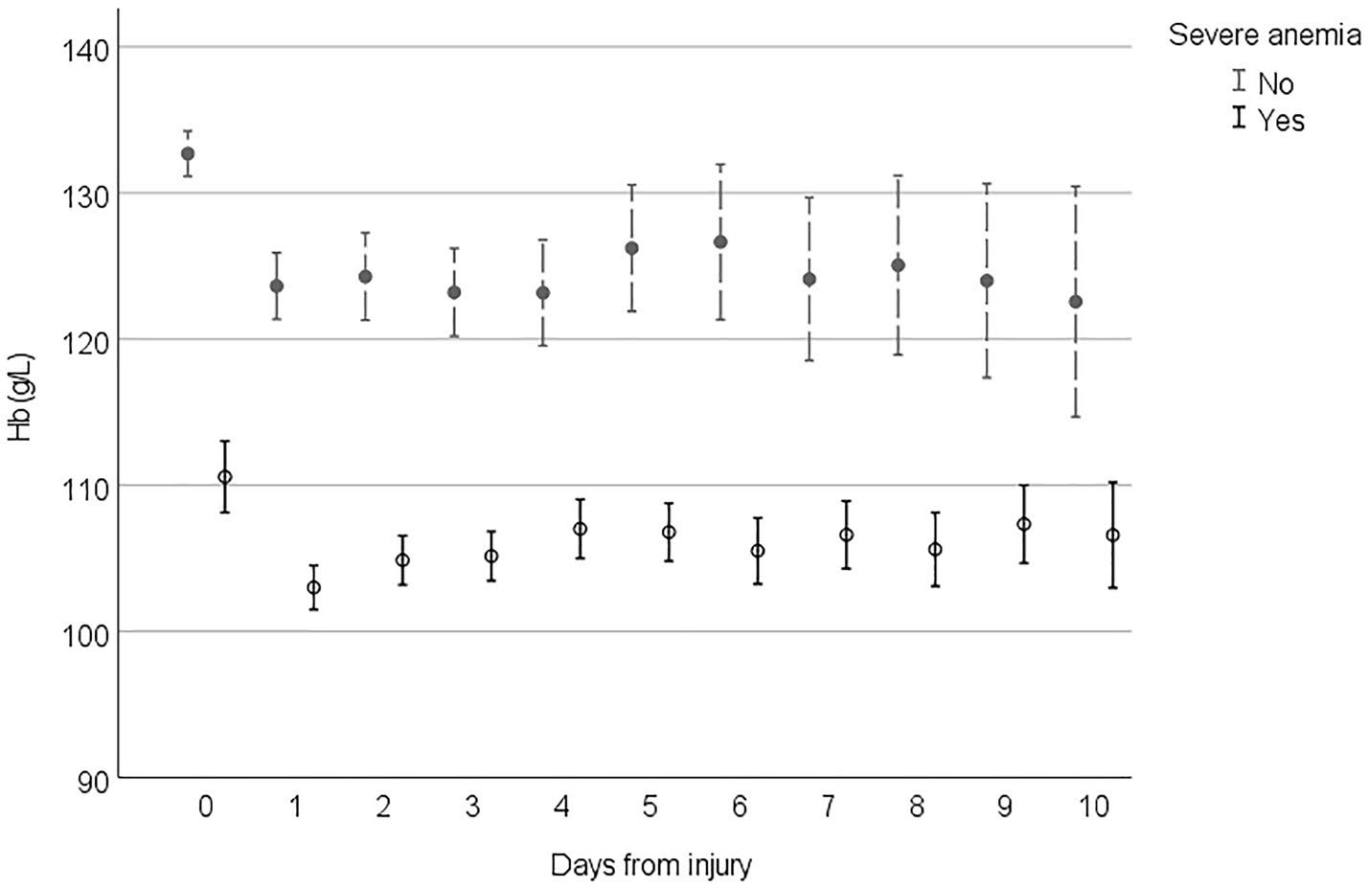
Hemoglobin (Hb) level trajectories in patients with traumatic brain injury with severe anemia (Hb level < 100 g/L) compared with nonanemic patients

**Fig. 3:**
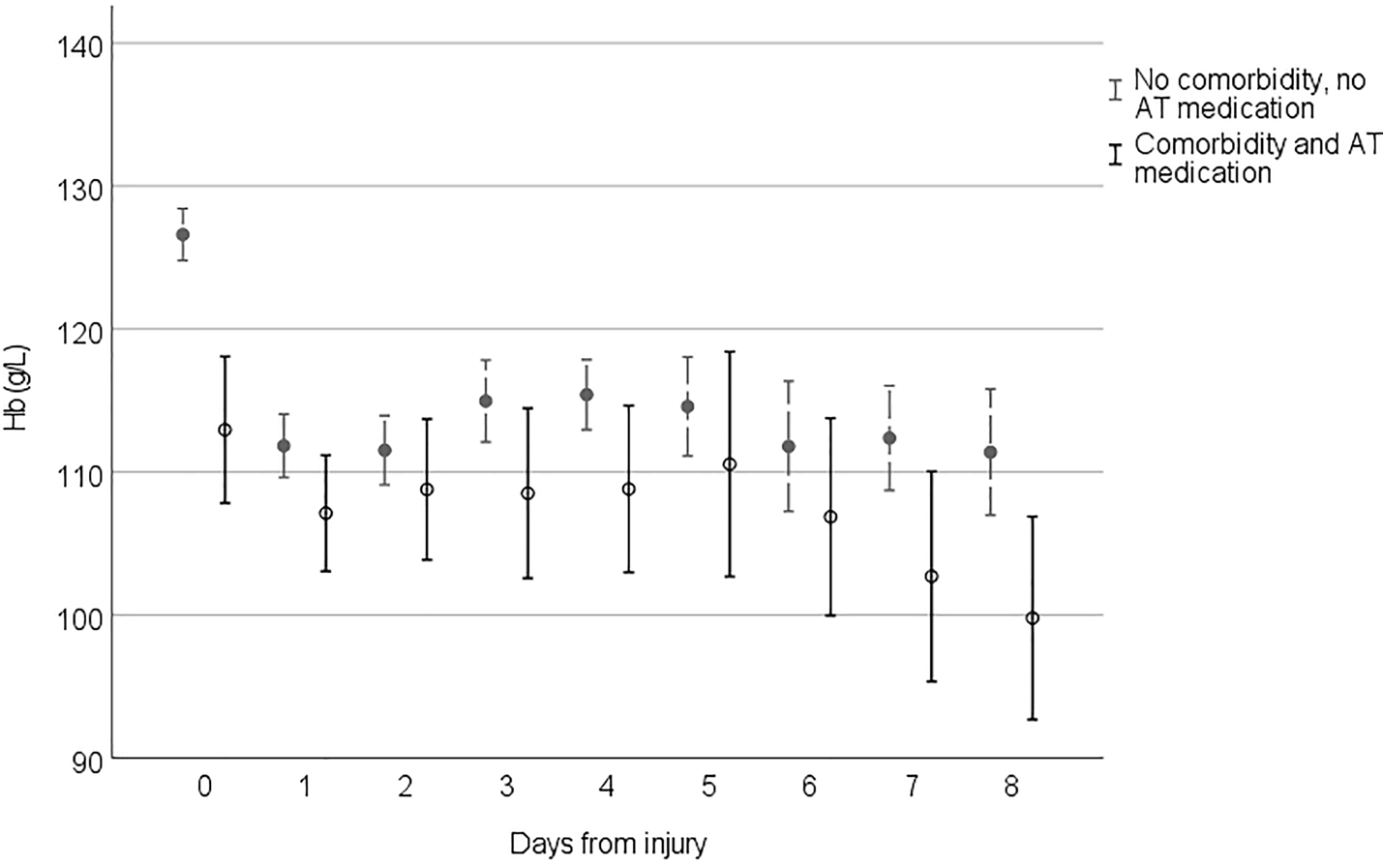
Hemoglobin (Hb) level trajectories in patients with moderate to severe traumatic brain injury with clinically significant comorbidities and antithrombotic medication compared with patients without them. *AT* antithrombotic

**Fig. 4:**
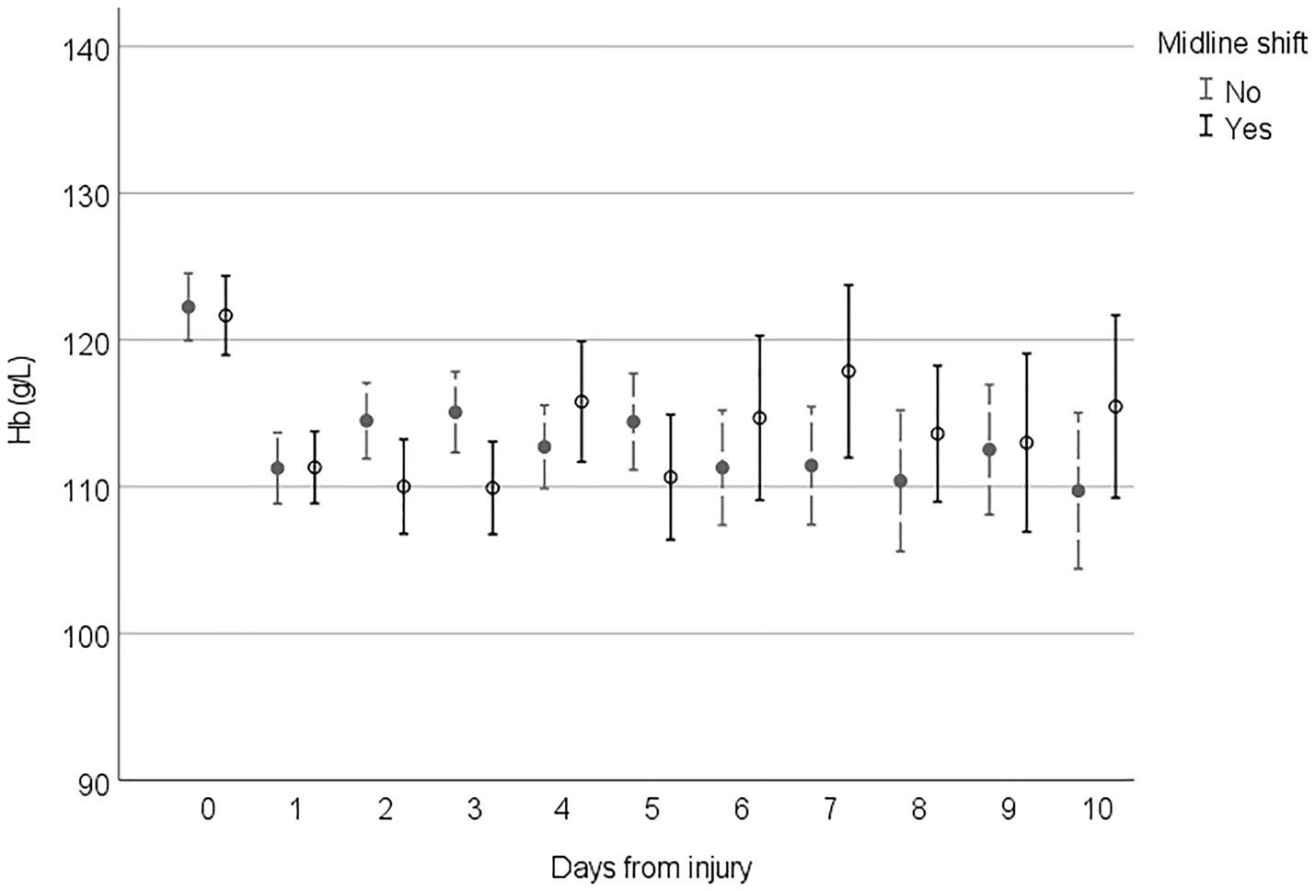
Hemoglobin (Hb) level trajectories in patients with moderate to severe traumatic brain injury with midline shift on primary head computed tomography

**Fig. 5:**
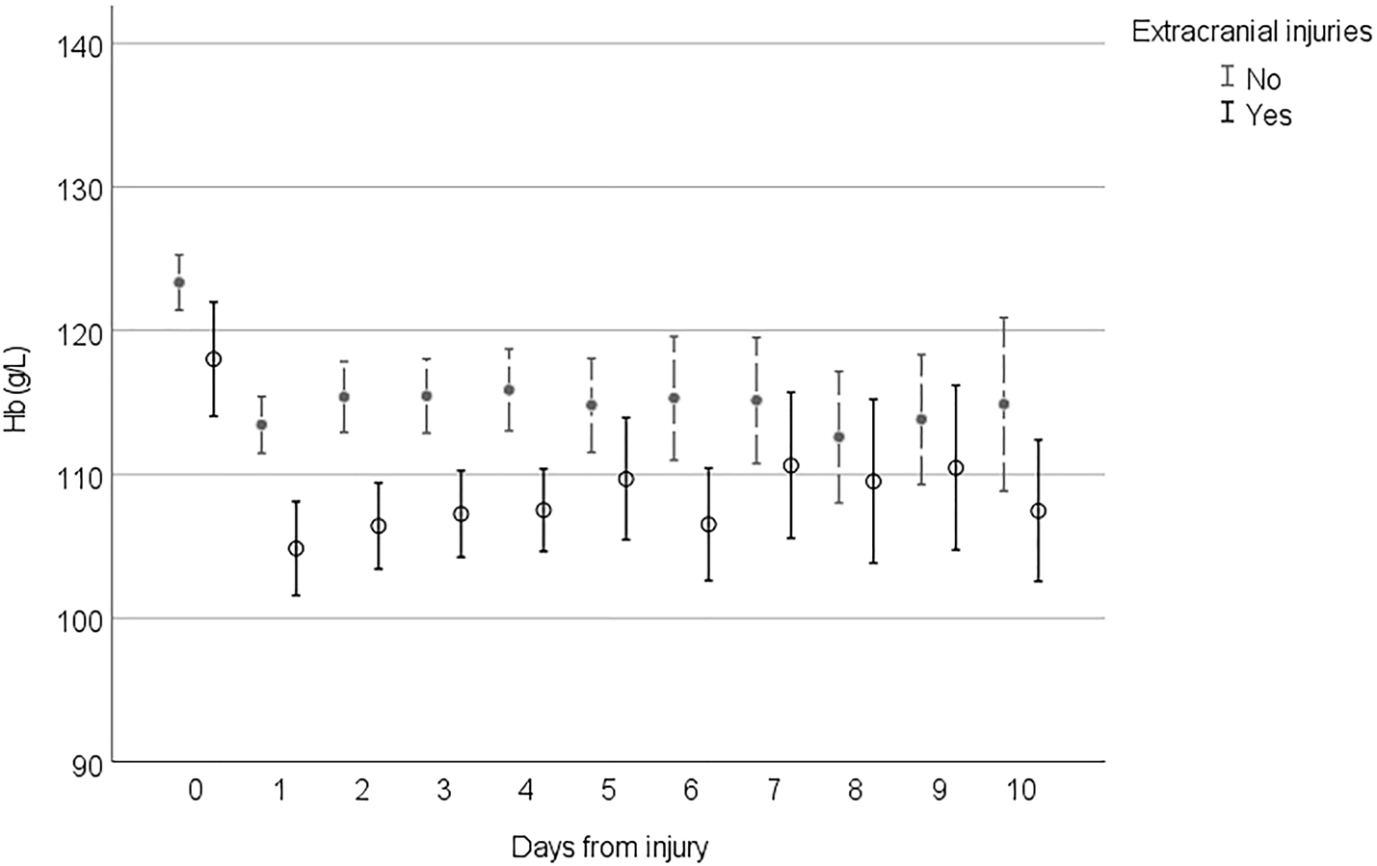
Hemoglobin (Hb) level trajectories in patients with moderate to severe traumatic brain injury with extracranial injuries

Figures 2, 3, 4 and 5 demonstrate the development of Hb values during the hospital stay in different patient groups, presenting the mean of the lowest measured Hb value of each 24-h time frame from the injury, the time of the insult being hour 0. In every studied subgroup, the deepest decline in Hb level was seen during the first 48 h after the trauma. Those with severe anemia had remarkably lower Hb levels constantly during the hospital admission (Fig. 2). Among patients with antithrombotic medication and major comorbidities, Hb levels were lower, and the decline during the first 48 h was more moderate than that in patients without them (Fig. 3). In this subgroup, Hb levels also tended to remarkably fall when the LOS was prolonged. Hb levels among patients with midline shift did not notably differ from those among patients without midline shift after the steep decline seen during the first 48 h (Fig. 4). In patients with extracranial injuries, the early decline in Hb level trajectory was considerably deeper than that among patients with an isolated TBI, and the difference lasted several days after the trauma (Fig. 5).

## Discussion

In our study, 45.5% of the patients developed severe anemia (Hb level less than 100 g/L) during hospitalization for acute moderate to severe TBI. According to WHO criteria, the incidence of anemia was almost 90%. The univariate analysis associated female sex, cardiovascular comorbidities, diabetes, and antithrombotic medication with severe anemia, as well as extracranial injuries, midline shift on admission CT, and major neurosurgical interventions. In the multivariable analysis, only midline shift and the presence of extracranial injuries resulted in a statistically significant association. Regardless of the subgroup, the greatest decline in Hb level was seen between 24 and 48 h after the trauma.

The incidence of severe anemia in our study is somewhat greater than that reported in the general ICU population [27, 28] but remarkably lower than that reported among patients in the surgical ICU [29]. The blood loss due to closed head trauma is relatively minute and is unlikely to cause a significant decrease in the Hb level alone, even when operated, which might explain the lower incidence of anemia compared with the general surgical ICU population. Instead, the development of anemia in patients with TBI is likely to be multifactorial, including possible hemodilution due to intravenous fluid resuscitation and bleeding (preoperative and perioperative) of extracranial traumas. Among patients with severe TBI and prolonged hospitalization, a late decrease in the Hb level is likely to resemble the anemia seen in the general critical care population during an extended LOS in the ICU, involving hemodilution, repeated phlebotomies, and inflammatory processes resulting in bone marrow suppression and impaired erythropoiesis [30]. The incidence of severe anemia in our study is in line with the results from a large retrospective study by Salim and colleagues [15], with a 46% incidence of severe anemia. However, there are some major methodological differences between the studies. The study population of Salim et al. [15] included all patients with blunt TBI admitted to the surgical ICU, regardless of the severity of the TBI, thus including also mild TBIs. In addition, anemia was defined as an Hb level less than 90 g/L for three consecutive samples, a definition markedly stricter than that in our study.

Reported data of factors associated with the incidence of anemia in patients with TBI are scarce. Salim et al. [15] did not investigate risk factors for anemia, but according to study population characteristics, anemic patients were more gravely injured, and the relative proportion of female patients was higher among anemic than nonanemic patients, findings in concordance with our study. Also, in line with our study, the large retrospective multicenter cohort study by Boutin et al. [31] demonstrated the association between female sex, comorbidities, extracerebral traumas, and RBCT, possibly indirectly indicating their association with anemia. In addition, the meta-analysis of RBCTs in patients with TBI indicated the association between RBCT and the severity of TBI [21]. The association between anemia and extracranial injuries seen in the multivariable analysis of our study is potentially related to bleeding from primary injuries and during surgery and fluid resuscitation for possible hypovolemia and hemodynamical instability. More excessive injuries might also lead to longer LOS in both the ICU and hospital, predisposing patients to anemia due to prolonged hospitalization as discussed earlier in this section. The role of midline shift in anemia is possibly associated with the same mechanisms: more severe trauma leads to longer hospitalization.

According to our univariate analysis, comorbidity, but not age, was associated with the development of anemia, suggesting that accumulation of chronic illnesses is more relevant than age-related fragility in development of acute anemia after TBI. This lack of association in the multivariable analysis might be at least partly related to overlapping of one or more subgroups: assumingly, patients with chronic (cardiovascular) diseases use antithrombotic medication more frequently than healthier patients, resulting in comparison of two groups with almost the same patients. We found no statistically significant association with the use of SSRI or SNRI medication and acute anemia in our study. The lack of association is likely related to the small proportion of the patients with continuous SSRI or SNRI medication and the relatively weak effect of the serotonin reuptake inhibitors on platelet function [32].

To our knowledge, the pattern of Hb level trajectories during hospitalization has not been previously described in patients with acute TBI. In every studied subgroup, the major decline in Hb level was seen during the first 48 h from the injury (days 0–1). After the first 48 h, Hb levels tended to stabilize or slightly increase in most subgroups, a phenomenon possibly explained by early surgery of intracranial and extracranial traumas and hemodilution after fluid resuscitation. If the LOS in the hospital was prolonged, Hb levels decreased in patients with major comorbidities. In this subgroup of patients, frailty can be one factor associated with the lingering decrease in Hb levels. The decline in Hb levels seen with prolonged stay in the ICU correlates with findings of previous studies in the general ICU population [28, 33]. Patients with a more severe TBI (based on midline shift on CT) had higher Hb levels subacutely (> 4 days post-TBI) compared with patients with a less severe injury on CT. This finding can possibly reflect more aggressive treatment of declined Hb levels in severely injured patients.

Our study has several limitations. Firstly, the retrospective single-center study setting might cause bias because of preferred treatment modalities and practices and reduce the generalizability to other hospitals and TBI populations. Secondly, excluding patients with an LOS in the study center of less than 48 h might lead to inclusion of patients with more severe traumas, and on the other hand, to exclusion of patients with fatal injuries, either intracranial or extracranial, and thus cause selection bias. Thirdly, the use of midline shift as a surrogate of more severe trauma can be controversary, possibly ignoring severe, bilateral intra-axial lesions. Fourthly, because of retrospective nature of the study, the timing and frequency of Hb measurements varied between patients, resulting in a less accurate and structured setting. Fifthly, the delay from the insult to the hospital admission varied between patients, causing possible confounding in the time-dependent development course of anemia. Sixthly, extracranial traumas were not classified because of type, location, or severity, leading to inaccuracy in susceptibility to bleeding. Lastly, the study did not include data of RBCTs, which are likely to affect the incidence and course of anemia.

## Conclusions

Severe anemia in patients with acute moderate to severe TBI was common, affecting nearly half of the patients at some point during the hospitalization. Possible associated factors included female sex, clinically significant comorbidities, and the use of antithrombotic medication. Severe anemia was more common in patients with extracranial traumas and midline shift on primary head CT as a marker of space-occupying TBI. The most severe decline in the Hb level was seen during the first 48 h after the trauma. However, further prospective research on anemia risk factors is needed to confirm the findings.
